# Exposure to Mobile Phone-Emitted Electromagnetic Fields and Human Attention: No Evidence of a Causal Relationship

**DOI:** 10.3389/fpubh.2018.00042

**Published:** 2018-02-23

**Authors:** Giuseppe Curcio

**Affiliations:** ^1^Department of Biotechnological and Applied Clinical Sciences, University of L’Aquila, L’Aquila, Italy

**Keywords:** radiofrequency, cognitive effects, behavior, brain functions, GSM, UMTS

## Abstract

In the past 20 years of research regarding effects of mobile phone-derived electromagnetic fields (EMFs) on human cognition, attention has been one of the first and most extensively investigated functions. Different domains investigated covered selective, sustained, and divided attention. Here, the most relevant studies on this topic have been reviewed and discussed. A total of 43 studies are reported and summarized: of these, 31 indicated a total absence of statistically significant difference between real and sham signal, 9 showed a partial improvement of attentional performance (mainly increase in speed of performance and/or improvement of accuracy) as a function of real exposure, while the remaining 3 showed inconsistent results (i.e., increased speed in some tasks and slowing in others) or even a worsening in performance (reduced speed and/or deteriorated accuracy). These results are independent of the specific attentional domain investigated. This scenario allows to conclude that there is a substantial lack of evidence about a negative influence of non-ionizing radiations on attention functioning. Nonetheless, published literature is very heterogeneous under the point of view of methodology (type of signal, exposure time, blinding), dosimetry (accurate evaluation of specific absorption rate-SAR or emitted power), and statistical analyses, making arduous a conclusive generalization to everyday life. Some remarks and suggestions regarding future research are proposed.

## Introduction

Nowadays almost the totality of human beings on the Earth is directly or indirectly exposed to the electromagnetic fields (EMFs) emitted by mobile phones, base stations, and other types of wireless communication technologies. This is a logical consequence of the fact that there are more mobile devices than living people on the planet: the estimate of total world population was around 7.44 billion at the end of 2017 (1), while the number of mobile devices was higher than 8.46 billion (2). Such increase in number of mobile phones brought many researchers in the past 20 years to manifest interest toward possible effects of radiofrequency (RF) and microwaves (MWs) on human brain. At the same time, also concerns about possible health effects were raised, so that the World Health Organization decided to start a dedicated health topic. Are all these concerns and worries scientifically relevant? Can we reach some conclusions about the possible effects of such non-ionizing radiations on brain functioning?

It is now well known that, due to the close proximity between radiofrequency source and the human brain, a discrete amount of RF EMFs is transferred through the skull and reaches the brain. This low-level non-ionizing EMFs absorption could potentially induce a physiological influence on cerebral functioning and cognitive–behavioral outcomes. Accordingly, several mechanisms of action have been proposed: from those based on the thermal processes (3, 4) to the ones hypothesizing non-thermal mechanisms, such as the modulation of membrane ionic channels for Na+ and K+ (5), the alteration of intracellular Ca2+ homeostasis (6), the increase in neuronal excitability (7, 8), or also the activation of cellular stress response (9, 10).

One of the most investigated cognitive outcomes is attention: starting from the very first studies in this field, multiple types of attentions (selective, sustained, or divided attention) have been seen as one of the potentially mostly influenced aspects of human brain functioning. In the past years, these studies have been extensively reviewed [e.g., (11)] as well as meta-analyzed [e.g., Ref. (12, 13)], thus allowing to outline some main conclusions, albeit not conclusive. Nonetheless, the scientific interest keeps growing, as reflected by the steady publication rate increase, also in the last years (14).

Here, the main findings obtained in the last 20 years of research with respect to mobile phone effects on human attention will be briefly reviewed and discussed. The focus will be on laboratory volunteer studies (i.e., provocation studies with, at least, a real and a sham condition), involving only mobile phone-like signals and aimed to investigate attentional performance as a main outcome. Although some of these studies had multiple outcomes (other cognitive indices or neurophysiological measures), only attentional performance results have been considered here. Literature search was conducted at the end of 2017 on both PubMed and WoS databases and covered the period between 1998 and 2017. Almost all the studies reported here have been conducted on healthy adults, while just a few of them investigated effects on adolescents or children and on patients (epileptics or idiopathic environmental intolerance to EMF [IEI-EMF] individuals). A summary of all included studies, with experimental characteristics (exposure features, blinding, sample, investigated domain, results) is reported in Table 1.

**Table 1:** Studies assessing attentional performance.

Paper	Exposure characteristics^a^	Blinding	Sample^b^	Attentional domain investigated, specific dependent measure, and moment of evaluation^c^	Results^d^
Freude et al. (15)	GSM phone, over the left ear, 916.2 MHz	Single blind	16 volunteers (all males)	Selective attention	No effect
	SAR_10g_, 0.88 W/kg			VMT, simple finger movement task	
	About 13 min			Assessed during exposure	

Freude et al. (16)	GSM phone, over the left ear, 916.2 MHz	Single blind	16 volunteers (all males)	Selective and divided attention	No effect
	SAR_10g_, 0.88 W/kg		16 volunteers (all males)	VMT (first study)	
				VMT, simple finger movement task and two-stimulus task (second study)	
	About 6 min (first study), about 15 min (second study)			Assessed during exposure	

Preece et al. (17)	Mobile phone copy over right ear	Double blind	36 volunteers (18 females)	Selective, sustained, and divided attention	Decrease in choice-reaction times (particularly in the analog condition)
	Simulated GSM signal, 915 MHz, SAR not reported (mean output power, 0.125 W)			Simple and choice reaction times, digit vigilance task	
	Analogue signal, 915 MHz, SAR not reported (output power about 1 W)			Assessed during exposure	
	About 25–30 min				

Koivisto et al. (18)	GSM phone, ~4 cm from left side, 902 MHz	Single blind	48 volunteers (24 females)	Selective and sustained attention	Decrease in simple reaction time and vigilance tasks; decrease of time needed in a mental arithmetic task; fewer errors in vigilance task
	SAR not reported (average output power 0.25 W)			Reaction time performance (12 tasks)	
	About 60 min			Assessed during exposure	

Haarala et al. (19)	GSM phone over left ear, 902 MHz	Double blind	64 volunteers (32 females)	Selective and Sustained attention	No effect
	SAR_1g_, 0.88 W/kg (peak 1.2 W/kg)			Cognitive functioning (9 tasks)	
Partial replication (18)	About 65 min			Assessed during exposure	

Jech et al. (21)	GSM phone, close to right ear, 900 MHz	Double blind	22 patients with narcolepsy–cataplexy (13 females)	Sustained attention	Decrease in reaction times
	SAR_10g_ 0.06 W/kg			Visual odd-ball paradigm	
	45 min			Assessed during exposure	

Edelstyn and Oldershaw (22)	GSM phone, hold by hand over left ear, 900 MHz	Single blind	38 volunteers	Selective, sustained, divided, and alternating attention	Improved performance at digit span forward, spatial span backward, and serial subtraction tasks
	SAR 1.19 W/kg (not directly measured)			Digit span forward/backward, spatial span forward/backward, serial subtraction, verbal fluency	
	30 min			Assessed before and after (15 and 30 min) exposure	

Croft et al. (23)	GSM phone, 5 cm from subject’s scalp between Oz and Pz, 900 MHz	Single blind	24 volunteers (8 females)	Selective attention	No effect
	SAR not reported (estimated average power 3–4 mW)			Auditory discrimination performance	
	20 min			Assessed under the exposure	

Lee et al. (24)	GSM phone, over right ear, 1,900 MHz	Single blind	78 volunteers (53 females)	Selective, sustained, and divided attention	Decrease in SART reaction times
	SAR not reported			Response Task (SART) and Trial Making Test-A and B	
	25 min			Assessed after the exposure	

Curcio et al. (25)	GSM phone, 1.5 cm from left ear, 902.4 MHz	Double blind	20 volunteers (10 females)	Selective, sustained, and divided attention	Decrease of both simple and choice reaction times
	SAR_10g_, 0.5 W/kg			Acoustic simple and choice reaction time task, visual search task, arithmetic descending subtraction task	
	45 min			Assessed both during and after the exposure	

Hamblin et al. (26)	GSM phone, over right temporal region, 894.6 MHz	Single blind	12 volunteers (8 females)	Sustained attention	Increase of reaction time
	SAR not reported (mean output power 0.25 W)			Visual odd-ball paradigm	
	60 min			Assessed during exposure	

Hamblin et al. (27)	GSM phone, over right or left ear, 895 MHz	Double blind	120 volunteers (74 females)	Sustained attention	No effect
	SAR_10g_, 0.11 W/kg			Auditory and visual odd-ball paradigm	
	30 min			Assessed after exposure	

Haarala et al. (28)	GSM phone, over left ear, 902 MHz	Double blind	32 children (16 girls)	Selective, sustained, and divided attention	No effect
	SAR_10g_, 0.99 W/kg			Reaction time performance (12 tasks)	
	~50 min			Assessed during exposure	

Preece et al. (29)	GSM phone, over left ear, 902 MHz	Double blind	18 children (9 girls)	Selective, sustained, and divided attention	No effect
	SAR 0.28 W/kg max in the brain (average output power 0.25 W)			Simple and choice reaction times, digit vigilance task [as in previous adult study (17)]	
	~30–35 min			Assessed during exposure	

Besset et al. (30)	GSM phone, over preferred ear, 900 MHz	Double blind	55 volunteers (EMF on: 14 females; EMF off: 13 males, 14 females)	Selective, sustained, and divided attention	No effect
	SAR_10g_, 0.54 W/kg			Simple reaction times, choice reaction times (2 versions), digit span forward, spatial span forward, modified Stroop task, figure cancelation test	
	120 min/day 5 days/week in 4 weeks			Assessed 4 times in a 45-day period	

Schmid et al. (31)	UMTS signal, close to left side, 1,970 MHz	Double blind	58 volunteers (29 females)	Selective and sustained attention	No effect
	SAR_10g_, 0.037, 0.37 W/kg			Critical Flicker and Fusion Frequency Test, Visual Pursuit Test, Tachistoscopic Traffic Test Mannheim, and Contrast Sensitivity Threshold	
	~60 min			Assessed during exposure	

Unterlechner et al. (32)	UMTS signal, close to left side, 1,970 MHz	Double blind	40 volunteers (20 females)	Selective, sustained attention	No effect
	SAR_10g_, 0.037, 0.37 W/kg			Simple reaction time, vigilance and determination tasks, Flicker and Fusion Frequency test	
	90 min			Assessed during exposure	

Keetley et al. (33)	GSM phone, 1.5 ± 0.5 cm from left ear, 900 MHz	Double blind	120 volunteers (62 females)	Selective, sustained, and divided attention	Impairment of simple and choice reaction times, and of sustained attention task
	SAR not reported (mean output power 0.23 W)			Simple reaction times, choice reaction times, digit span, Digital Symbol Substitution Test, Trail Making Task, Inspection time	Improvement of task switching/divided attention
	About 90 min			Assessed during exposure	

Wilén et al. (34)	GSM test phone, 8.5 cm from right side, 900 MHz	Single blind	20 volunteers with IEI-EMF (4 females)	Selective and sustained attention Critical Flicker Fusion Threshold	No effect
	SAR_10g_, 0.8 W/kg		20 healthy controls (4 females)	Assessed during exposure	
	30 min				

Russo et al. (35)	GMS and CW signal, over right (*n* = 42) or left (*n* = 42) ear, 888 MHz	Double blind	168 volunteers (99 females) half exposed to GSM and half to CW signal	Selective, sustained and divided attention	No effect
	SAR_10g_, 1.4 W/kg			Simple and choice reaction time task, subtraction task and vigilance task	
	~35- to 40-min per side			Assessed during exposure	

Haarala et al. (36)	Pulsed and CW signal, over right or left ear, 902 MHz	Double blind	36 volunteers (all males)	Selective, sustained, and divided attention	No effect
	SAR_10g_, 0.74 W/kg, peak 1.18 W/kg			Simple reaction times, 10 choice reaction time, subtraction, verification and vigilance tasks	
	~45 min per side			Assessed during exposure	

Terao et al. (37)	Pulsed EMF signal, over right ear, 800 MHz	Double blind	16 volunteers (23–52 years; 9 males, 7 females)	Selective and sustained attention	No effect
	SAR_10g_, 0.05 ± 0.02 W/kg (30 mm under scull)			Visuomotor choice reaction time, movement time and accuracy	
	30 min			Assessed before and after exposure	

Fritzer et al. (38)	GSM signal, three antennas 30 cm from head’ vertex, 900 MHz	Single blind	EMF on: 10 volunteers (all males)	Selective and divided attention	No effect
	SAR_1g_, 0.875 W/kg		EMF off: 10 volunteers (all males)	Trail Making Test-B, Attention stress test (d2)	
	8 h × 6 nights			Assessed before and after exposure	

Regel et al. (39)	GSM PM and CW signal, antennas 115 mm from left side, 900 MHz	Double blind	16 volunteers (all males)	Selective and sustained attention	No effect
	SAR_10g_, 1 W/kg			Simple and choice reaction times tasks	
	30 min			Assessed during exposure	

Regel et al. (40)	GSM signal, antennas 115 mm from left side, 900 MHz	Double blind	15 volunteers (all males)	Selective and sustained attention	No effect
	SAR_10g_, 0.2, 5 W/kg			Simple and choice reaction times tasks	
	30 min			Assessed during exposure	

Curcio et al. (41)	GSM phone, 1.5 cm from right ear, 902.40 MHz	Double blind	24 volunteers (12 females)	Selective and sustained attention acoustic simple reaction time task	No effect
	SAR_10g_, 0.5 W/kg			Assessed after exposure	
	15 min × 3 times				

Kleinlogel et al. (42)	GSM base station-like signal, antenna over left ear, 900 MHz	Double blind	15 volunteers (all males)	Selective and Sustained attention Continuous Performance Test	Increased errors in UMTS lowest level in one of two task conditions
	SAR_10g_, 1.0 W/kg				
	UMTS handset-like signal, 1,950 MHz			Assessed during exposure	
	SAR_10g_, 0.1, 1.0 W/kg				

Stefanics et al. (43)	UMTS mobile phone, antenna over right ear (frequency not specified)	Double blind	36 volunteers (20 females)	Sustained attention	No effect
	SAR_1g_, 0.39 W/kg			Auditory oddball paradigm	
	20 min			Assessed before and after exposure	

Riddervold et al. (44)	TETRA handset, over left side, 420 MHz	Double blind	53 emergency service personnel (all males)	Selective and divided attention	No effect
	SAR_10g_, 2.0 W/kg			Simple reaction times, Trail Making Test-B	
	45 min			Assessed during exposure	

Kwon et al. (45)	GSM phone, over right ear, 902.4 MHz	Double blind	13 volunteers (all males)	Selective and sustained attention	No effect
	SAR_10g_, 0.7 W/kg			Simple visual vigilance task	
	33 min			Assessed during exposure	

Kwon et al. (46)	GSM phone, over right ear, left ear and forehead, 902.4 MHz	Double blind	15 volunteers (all males)	Selective and sustained attention	No effect
	SAR_10g_, 0.7 W/kg (right), 1.0 W/kg (left), 0.7 W/kg (forehead)			Visual vigilance task	
	5 min, 3 times for each condition			Assessed during exposure	

Sauter et al. (47)	GSM signal, 900 MHz or UMTS signal, 1,966 MHz, antenna over head	Double blind	30 volunteers (all males)	Selective, sustained, and divided attention	No effect
	SAR_10g_, 2 W/kg			Test for attentional performance, sustained attention from Vienna System testing	
	About 7 h 15 min per day, each condition on 3 days			Assessed during exposure	

Curcio et al. (48)	GSM phone, 1.5 cm from right ear, 902.40 MHz	Double blind	12 volunteers (all males)	Selective and sustained attention	No effect
	SAR_10g_, at 2 cm depth 0.5 W/kg			Go–No Go task	
	45 min			Go-No Go task	

Schmid et al. (49)	PM signal, antenna 115 mm from left side, 900 MHz (PM at 14 and 217, respectively)	Double blind	30 volunteers (all males)	Selective and Sustained attention	No effect
	SAR_10g_, 2 W/kg			Simple reaction time task, 2 choice reaction time task	
	30 min			Assessed during exposure	

Schmid et al. (50)	PM RF signal, antenna 115 mm from left side, 900 MHz (PM at 2 Hz)	Double blind	25 volunteers (all males)	Selective and sustained attention	Improved speed in one task (only under PM signal)
	SAR, 2 W/kg			Simple reaction time task, 2 choice reaction time task	
	Pulsed magnetic field, Helmholtz coils at both sides, pulse frequency 2 Hz			Assessed during exposure	
	Peak magnetic flux density 0.70 mT, 30 min				

Loughran et al. (51)	GSM-like signal, antenna on left side, 900 MHz	Double blind	22 volunteers (10 females)	Selective and sustained attention	No effect
	SAR_10g_ 1.33 W/kg, 0.35 W/kg			Simple reaction time task, 2 choice reaction time task	
	30 min			Assessed during exposure	

Trunk et al. (52)	UMTS mobile phone, 4–5 mm from right ear, 1,947 MHz	Double blind	21 volunteers (9 females)	Sustained attention	No effect
	SAR_1g_, 1.75 W/kg (at 20 mm in depth)			Visual odd-ball paradigm	
	15 min			Assessed previous, during and after exposure	

Trunk et al. (53)	UMTS mobile phone, 4–5 mm from right ear, 1,947 MHz	Double blind	23 volunteers (13 females)	Selective attention	No effect
	SAR_1g_, 1.75 W/kg (at 20 mm in depth)			Target probability processing	
	15 min			Assessed previous, during and after exposure	

Eggert et al. (54)	TETRA signal, antenna 10 mm from skin at left side, 385 MHz, PM at 17.65 Hz	Double blind	30 volunteers (all males)	Selective and sustained attention	No effect
	SAR_10g_, 1.5 W/kg (low), 6 W/kg (high)			Clock visual monitoring task	
	150 min in each of 3 sessions for each condition			Assessed immediately before and after each exposure	

Sauter et al. (55)	TETRA signal, antenna 10 mm from skin at left side, 385 MHz, PM at 17.65 Hz	Double blind	30 volunteers (all males)	Selective, sustained, and divided attention	Reduced variability of speed at vigilance task under low TETRA
	SAR_10g_, 1.5 W/kg (low), 6 W/kg (high)			Test for attentional performance, Sustained attention from Vienna System testing	
	150 min in each of 3 sessions for each condition			Assessed before and after each exposure	

Malek et al. (56)	GSM 900 and 1800, and UMTS signal, antenna at 2 m from subjects, 945 MHz 1840 and 2,140 MHz, respectively	Single blind	200 volunteers (100 with IEI-EMF and 100 non-IEI-EMF)	Selective and sustained attention	No effect
	SAR not reported (power flux density 280, 250, and 380 W/m^2^, respectively)			Reaction times, Rapid Visual Processing task, spatial span	
	Exposure duration not reported			Assessed before and after each exposure	

Verrender et al. (57)	GSM signal, 1.15 cm from both ears, 920 MHz	Double blind	36 volunteers (18 females)	Selective attention	No effect
	SAR_10g_, 1 W/kg (low), 2 W/kg (high)			Visual discrimination task	
	30 min			Assessed during or after exposure	

Altuntas et al. (58)	GSM phone, hold over left ear, 900–1,800 MHz	Double blind	30 volunteers (11 females)	Selective and sustained attention	Improvement of measure of accuracy (limited to selective attention)
	SAR not reported			D2 test of attention and concentration	
	15 min			Assessed before and after exposure	

*^a^Exposure features are derived from information reported in the article*.

*^b^Participants were always healthy adults, if not differently specified*.

*^c^Only outcomes related to attention are reported and discussed*.

*^d^Only significant effects are summarized*.

*EMF, electromagnetic field; IEI-EMF, idiopathic environmental intolerance to EMF*.

## Literature Findings

In the first study investigating the effects of MW emissions on the brain, visual monitoring was also tested (15). In a single-blind study, participants were exposed for about 13 min to a GSM signal. No significant effects were reported on attentional performance. The same group (16) replicated and extended the study 2 years later by using several tasks assessing attentional performance. Also in this single-blinded study, no significant effects were reported on volunteers’ performance.

Preece et al. published one of the first studies on attention and vigilance (17). The authors were interested in the effects of a simulated mobile phone signal at 915 MHz in healthy adults. Participants were tested in 10 different tasks following 25- to 30-min of exposure with double-blind administration. The only significant effect reported was a reduction in choice reaction times, while no relevant effects were seen on any of the other nine tasks.

Koivisto et al. (18) with a single-blind procedure investigated the effects of mobile phone exposure on response times to 12 different tasks. After 60 min of exposure, a significant improvement of vigilance and attention was reported. Some years later, Haarala et al. (19) extended and methodologically improved this study (double-blind design, larger sample size, multicentre testing, some additional tasks): after 65 min of exposure to the same signal used in the previous study, no significant effects were reported on attention tasks. A similar inconsistency was also highlighted by the same authors when they tried to replicate similar studies [see, for example, Ref. (20)].

Jech et al. (21) for the first time investigated the cognitive effects of mobile phone exposure in a sample of patients with narcolepsy–cataplexy. After 45 min of GSM exposure, the participants were asked to complete a visual odd-ball paradigm for the evaluation of sustained attention. The authors reported a facilitating trend on reaction times, while no effects for accuracy were observed.

A similar effect to the one evidenced by Koivisto et al. (performance speeding up and attentional capacity improvement) was reported by Edelstyn and Oldershaw (22) after only 5 min of single-blind exposure to a GSM signal on two tests of attentional capacity (of six administered).

Croft et al. (23) investigated the influence of mobile phone exposure on neural functioning, including performance in an auditory discrimination task. During the 20-min exposure to GSM signal, participants completed the discrimination task four times: no significant differences were observed on performance.

In a study on university students, performance at different tasks was assessed, including an auditory vigilance test (24). Participants in the experimental group performed better on this test only after they had been exposed to the GSM EMFs, thus suggesting an improvement of participants’ attention.

Curcio et al. (25) exposed the participants to GSM for 45 min, both before and during the cognitive testing. Results indicated a faster performance to both simple and choice reaction time tasks when the field was “on” with respect to the sham condition: such a speeding up of performance was more evident after at least 25 min of exposure to the signal.

Hamblin et al. (26) studied the effects of mobile phone exposure on psychomotor performance during an auditory task. Here, participants were exposed to a GSM and sham signal for 60 min: a reduced speed under real exposure with respect to placebo condition was reported. Two years later, the same group, with a similar setting but with substantial methodological improvement (i.e., double blinding) exposed their participants to a signal on both side of the head (27). Results indicated no significant differences on attentional performance.

In 2005, Haarala et al. (28) carried out the first experiment on children aiming at assessing the effects of MP exposure on cognitive performance. Several tasks were selected to evaluate different aspects of attention: no statistically significant differences between sham and real conditions were observed on cognitive functioning. A further experiment on adolescents was independently carried out by Preece et al. (29). The authors investigated the effect of EMFs by means of participant’s response times to attentional tasks: again, no significant effects were reported.

In a unique effort to simulate a real-life exposure, Besset et al. (30) planned a complex and long protocol of exposure, lasting 45 days (3 of baseline, 28 of exposure period, 14 of recovery) during which volunteers were exposed 2 h per day, 5 days per week. Despite the use of several attentional outcomes, no impact of exposure was highlighted.

Schmid et al. (31) in a methodologically sound study investigated the effects of the exposure to a third-generation mobile phone (UMTS) on visual perception as assessed by means of four different perceptual attention tasks. No significant differences were reported on indices of both speed and accuracy in any of the four tasks used. Some years later, the same group (32) carried out a companion study with the same exposure system and conditions, to assess the effects of low and high intensity of UMTS exposure compared to sham condition, on attention and reaction time tasks. Again, no significant differences were reported on indices of both speed and accuracy in any of the four tasks used.

Keetley et al. (33) aimed at investigating the effect of exposure to GSM signal on some different cognitive tasks administered to a large sample of 120 volunteers, controlled for age, education, and gender. They reported very inconsistent results: an impairment of performance in a simple- and a choice-reaction time task and in a sustained attention task and a contemporary improvement in switching abilities and divided attention as a consequence of an exposure to GSM-like signal lasting 90 min.

The first study on individuals with IEI-EMF was carried out with a single-blind protocol, exposing participants for 30 min (34). No effects were observed on a basic arousal/vigilance task as a consequence of the exposure.

Similar to other studies that attempted to control for methodological limitations (small sample size, single-blind design, type of exposure signal) of previously published studies, Russo et al. (35) enrolled 168 volunteers to investigate the effect on attention of 35- to 40-min exposure to a pulsed (GSM-like), CW, and sham signal. Again, no effect of exposure on measures of attention and speed processing was reported. Independently, Haarala et al. (36) conducted a study with analogous setup and methodology. As in the Russo et al.’s study (35), no effect of exposure to real signal on attentional tasks has been reported.

Terao et al. (37) investigated motor preparation performance before and after a 30-min exposure to a GSM signal. Also in this double-blind study, no effects were observed on measures of accuracy, reaction time, or speed as a function of exposure to the EMF.

Fritzer et al. (38) conducted a single-blind study investigating short- and long-term effects of RF EMF exposure on attentional functions after multiple nighttime exposures to GSM signal. No effects on neuropsychological tests were reported as a function of exposure to the field.

To disentangle the differential effects of different signals (pulsed GSM, CW, sham), an experimental study was carried out to assess the effects of 30 min of irradiation on several attentional tasks (39). Results indicated no significant effects as a function of the presence of real signal. Another study by the same group (40) aimed at investigating possible dose-dependent effects of GSM signals on attention tasks. After an exposure of 30 min, again no significant performance changes between real and sham condition have been reported.

In 2008, a study attempted to test the possible cumulative effects of brief (15 min) and repeated (three times) exposures in a single daily session (41). By using an exposure setting identical to the previous work (25), no statistically significant difference arose as a consequence of exposure to the GSM radiation on attentional performance.

Another study directly aiming at comparing the possible effects of GSM and UMTS signals was proposed by Kleinlogel et al. (42). Following a double-blind design, participants were exposed for 30 min to different conditions (GSM; UMTS “low” intensity; UMTS “high” intensity). No significant effects were observed for vigilance task, while a slight increase in errors was seen under lowest level of UMTS signal, limitedly to one task.

In a study mainly aimed at investigating EEG features during an auditory oddball paradigm, Stefanics et al. (43) exposed 36 participants to a 3G UMTS and sham signal for 20 min. Performance (accuracy index) was tested before and after exposure: also in this case, no statistically significant effects of exposure were reported.

To investigate the impact of TETRA signals on cognitive function, Riddervold et al. (44) exposed emergency service personnel for 45 min. The signals were generated by a TETRA handset connected to an external antenna placed in the “cheek position.” No changes on attention were reported as a function of TETRA exposure.

In a high-resolution PET study, vigilance was assessed after 33 min of GSM exposure (45): again, no effect of exposure was observed on both speed and accuracy measures. A companion study by the same research group aimed at comparing different exposure conditions each lasting 5 min (46). Exposure, independently by irradiated region and emitted power, was not found to have any influence on attentional task performance.

In the same year, another attempt to compare possible cognitive effects of GSM and 3G UMTS signals was carried out (47). Three tasks measuring different types of attention were administered, and none of them showed statistically significant differences between real and sham condition.

In an fMRI study, attentional performance was assessed after 45 min of GSM exposure by means of a somatosensory Go-No Go task (48). No exposure-related effects on accuracy or speed of attention were reported.

In the same vein of previously discussed Regel et al.’s studies, Schmid et al. (49) exposed their participants for 30 min before sleep to differently modulated GSM signals. Again, no statistical evidence of an influence of EMFs signals on the attentional performance was observed. In a second study by the same group (50), volunteers were exposed to both a pulse-modulated RF signal and a pulsed magnetic field for 30 min prior to a full night’s sleep: here an attention task was completed both during the first and the last 15 min of exposure. Results indicated a worsening in performance speed, only under pulsed magnetic field exposure.

Recently, another study on adolescents was carried out (51) comparing mobile phone-like RF EMFs at two different intensities with a sham session. Some cognitive tasks were performed during the 30-min exposure period; also in this study, no effect of exposure on a simple reaction time task was observed.

Trunk et al. (52) investigated the effects of 3G UMTS mobile phone exposure and caffeine consumption on attention. In a double-blind design, participants underwent different exposure conditions (Sham, Caffeine; Mobile phone; Caffeine and mobile phone). Although caffeine showed clear and expected effects on the RT, no effect of mobile phone exposure was reported neither alone nor in combination with caffeine. One year later, the same group conducted a companion study with the only difference that performance was assessed by means of a different task (53). Also in this case, no effect of signal exposure was reported, and no effects were seen when exposure was combined with caffeine.

Two associate studies tested the effects of two levels of TETRA-like exposure (“low” and “high”) on volunteers exposed for 150 min (54, 55). In the first study (54), the same attentional task of Freude et al. (15, 16) was used and no effect of exposure was observed on performance outcomes. The other study (55) investigated different aspects of attention by means of different tasks, and only a slight effect (1 of 35 parameters assessed) was reported. In this case, variability of speed measure at vigilance task resulted decreased under “low” TETRA exposure with respect to the other conditions.

Recently, a single-blinded study was carried out to test the effect of both GSM and UMTS on a considerably large sample (56) composed of both IEI-EMF individuals and healthy controls. Also in this case, no effects on attention were observed as a consequence of this short-term exposure to the signals.

Verrender et al. (57) aimed at investigating the effects of pulse modulated GSM signal on cognition and possible dose-dependent influences. By using two different levels of peak-spatial SAR (low and high RF), no effect has been found on visual discrimination performance, neither at low nor at high level of exposure.

Finally, a recent study was conducted on emergency physicians (58) to test the effects of an acute exposure to mobile phones on their attention. Participants were randomly assigned to an exposed group or to a non-exposed group, and attentive performance was assessed before and after the acute irradiation. Results indicated a positive impact on levels of selective attention as a consequence of a brief and acute exposure to mobile phone EMF.

## Discussion

The first studies on humans addressing attentional performance changes as a consequence of RF EMFs exposure date back to almost 20 years ago. In this mini-literature review, of 43 studies, 31 indicated the absence of statistically significant differences between real and sham signal, 9 showed changes in the direction of a partial improvement of attentional performance as a function of real exposure, while the remaining three showed very mixed and contrasting results (see Table 1 for details). This qualitative analysis overlaps the one proposed some years ago by Kwon and Hämäläinen (11) and is consistent with quantitative-based conclusions reached in two different meta-analyses (12, 13), both pointing to a lack of consistency in short-term acute effects of GSM-EMFs exposure on attentional domains. More generally, most of the poorly controlled studies, with relatively low methodological robustness (i.e., single blinding, limited sample size) and limited attention to exposure aspects (no control for dosimetry or emitted power) reported a better performance following mobile phone exposure. These effects basically disappeared when sample size and methodology were controlled for (see below). Thus, despite the public concern about potential biologic effects of acute RF EMFs, it can be concluded that there is a substantial lack of evidence of the influence of low-energy non-ionizing radiations on one of the major measures of cognitive functioning, namely attention.

Unfortunately, these conclusions are undermined by the extreme heterogeneity of available literature. If we look at the published papers (see Table 1), in fact, we can find studies using different signals (GSM, UMTS, TETRA), very different emitted powers and/or SARs, released by different antennas (planar, linear, internal to the phone), carried out on different samples (healthy adults, adolescents, children, patients), with different exposure duration (varying from a few minutes to several hours per day) and methodological settings [see also (12, 13)].

Looking at the available literature, blinding procedure and sample size seem to crucially affect studies outcomes. Several effects observed and reported in the very first studies (with low experimental control and not ever fully blinded) have not been replicated in more methodologically sound studies (usually double-blinded): this is the case, for example, of the pioneering study by Koivisto et al. (18) who showed a speeding up of performance, an effect not confirmed when the study was extended and methodologically improved (19).

The same happened when sample size was controlled for: in some cases, the effect previously shown on sustained attention measures [e.g., Ref. (26)] was not confirmed when a very similar protocol was applied to an adequately enlarged sample [e.g., Ref. (27)].

Other very central methodological issues are related to both a correct dosimetry assessment and a comparability among different experimental settings. As shown in Table 1, when an appropriate evaluation of SAR is reported, results are most likely to be negative. Conversely, when dosimetric data are lacking (17, 18, 24) or incomplete [providing only emitted power, i.e., Ref. (22, 26, 33)], some positive results can be highlighted. Furthermore, SAR and/or output power are still very varying between studies, reducing the possibility of a direct comparison between exposure setting and cognitive effects.

If we take into consideration all these differences in methodology and experimental setting, it becomes very difficult to come to a general conclusion on potential everyday life effects.

On the basis of the present systematic review of literature, some future research issues can be outlined as well as aspects that would merit further investigation.

As a first, it is mandatory to control for procedural aspects, by exposing participants for similar or equivalent periods of time: a rapid look at Table 1 shows that duration of exposure are very heterogeneous and usually very limited (around 30–45 min per day). In this way, only the effects of very acute and “short” irradiations can be explained, while no details on a more ecologic exposure (some hours per day) can be obtained.

Linked to the previous issue is the one of medium- and long-term effects to repeated EMFs exposure: this is a very relevant exposure procedure because it mimics very well what happens in the real life. To date, only one study (30) tried to do a medium-term study with a relevant amount of daily exposure time (120 min/day 5 days/week in 4 weeks). Up to date, no studies on long-term effects (months or years) have been carried out.

Even related to methods is the topic of sample characteristics. As stressed in previous reviews [e.g., Ref. (12)], individuals in a critical developmental period (such as infants and/or adolescents) need to be investigated because their not completely matured brain could be influenced by these kind of radiations. In the last years, some attempts have been done [e.g., Ref. (28, 29, 51)], particularly on adolescents and preteenagers, but more attention should be dedicated to the youngest. In the same vein, more studies should be done on potentially sensitive populations such as elderly or neurological impaired patients, also to verify some hypotheses put forward in the last years on animal models about a potential selective benefit at cognitive level [e.g., Ref. (59, 60)] and recently extensively reviewed and discussed (61).

Finally, some more research should be carried out “on the field,” namely on individuals that are exposed to RF EMF for working reasons [as in Ref. (44, 54)]. This would not only show if a constant exposure to these kind of signals can influence cognitive abilities of emergency service employees but also shed further light on possible health complaints in this type of workers.

## Concluding Remarks

On the basis of reviewed literature, we can reasonably conclude that there is no evidence of a negative influence of mobile phone emitted EMFs on different aspects of human attention. As pointed out in Discussion, published literature is very heterogeneous with respect to methodology, dosimetry, or statistical analyses, and thus a conclusive generalization to everyday life is still very difficult. For these reasons, further research is needed, particularly on real-working settings and environments.

## Author Contributions

GC conceived of, wrote, and edited this review.

## Conflict of Interest Statement

The author declares that the research was conducted in the absence of any commercial or financial relationships that could be construed as a potential conflict of interest.
